# Communicating the Neuroscience of Psychopathy and Its Influence on Moral Behavior: Protocol of Two Experimental Studies

**DOI:** 10.3389/fpsyg.2017.00294

**Published:** 2017-03-14

**Authors:** Robert Blakey, Adrian D. Askelund, Matilde Boccanera, Johanna Immonen, Nejc Plohl, Cassandra Popham, Clarissa Sorger, Julia Stuhlreyer

**Affiliations:** ^1^Centre for Criminology, University of OxfordOxford, UK; ^2^Department of Psychology, University of OsloOslo, Norway; ^3^Department of Psychology, King’s College LondonLondon, UK; ^4^Psychology Unit, University of HelsinkiHelsinki, Finland; ^5^Department of Psychology, University of MariborMaribor, Slovenia; ^6^Department of Experimental Psychology, University of OxfordOxford, UK; ^7^Division of Psychology and Language Sciences, University College LondonLondon, UK; ^8^Department of Psychology, Leiden UniversityLeiden, Netherlands

**Keywords:** psychopathy, belief in free will, utilitarian moral judgment, neuroscience communication, dishonesty, attributions, belief in determinism, self-control

## Abstract

Neuroscience has identified brain structures and functions that correlate with psychopathic tendencies. Since psychopathic traits can be traced back to physical neural attributes, it has been argued that psychopaths are not truly responsible for their actions and therefore should not be blamed for their psychopathic behaviors. This experimental research aims to evaluate what effect communicating this theory of psychopathy has on the moral behavior of lay people. If psychopathy is blamed on the brain, people may feel less morally responsible for their own psychopathic tendencies and therefore may be more likely to display those tendencies. An online study will provide participants with false feedback about their psychopathic traits supposedly based on their digital footprint (i.e., Facebook likes), thus classifying them as having either above-average or below-average psychopathic traits and describing psychopathy in cognitive or neurobiological terms. This particular study will assess the extent to which lay people are influenced by feedback regarding their psychopathic traits, and how this might affect their moral behavior in online tasks. Public recognition of these potential negative consequences of neuroscience communication will also be assessed. A field study using the lost letter technique will be conducted to examine lay people’s endorsement of neurobiological, as compared to cognitive, explanations of criminal behavior. This field and online experimental research could inform the future communication of neuroscience to the public in a way that is sensitive to the potential negative consequences of communicating such science. In particular, this research may have implications for the future means by which neurobiological predictors of offending can be safely communicated to offenders.

## Introduction

Since the time of Aristotle it has been argued that all human behavior can be described in terms of deterministic causality, and that there is no such thing as free will. Although philosophical arguments challenging free will have existed for centuries, these arguments do not appear to have filtered into the lay mind. However, there has been much recent lay interest in the rise of neuroscience as a means of explaining complex behaviors (Legrenzi and UmiltaÌ, 2011). Therefore, one might predict that in the future, lay belief in free will could be challenged through the communication of neuroscience. Hence empirical research has begun to test whether people believe that free will could exist in a world where all events were products of brain activity (Nahmias et al., 2007).

This study is concerned with the behavioral implications of such beliefs. One of the greatest consumers of the neuroscience behind behavior could be people who might benefit from a neurobiological understanding of their mental condition. People may be especially receptive to neuroscience if the explanation is construed as a scientific means of excusing the socially disapproved symptoms of their condition. One such condition is psychopathy. Psychopaths have been shown to differ from ordinary people in both neurobiological and cognitive terms. For example, previous research has shown that psychopaths differ from lay people in moral dilemmas, such that they choose utilitarian reasoning more often. One focus of our study is therefore whether a neuroscientific explanation of typical psychopathic behavior will affect behavior in this sort of task, perhaps by excusing the behavior as not a result of free will.

In our field study, we will test whether lay people are more likely to return a postcard that contains a cognitive rather than a neurobiological explanation of criminal behavior, and whether they are more likely to return the postcard when it is directed to prisoners or non-prisoners. Subsequently, we will conduct an online study in which participants will be given false feedback about having above-average or below-average psychopathic traits; we are investigating the effects of communicating either a neurobiological or cognitive explanation of psychopathy on reasoning in moral dilemmas and behavior in a measure of actual cheating (Shalvi et al., 2012).

### Behavioral Attributes of Psychopaths – the Lack of Empathy and Utilitarian Reasoning

Most established definitions of psychopathy emphasize two main characteristics of psychopaths: emotional impairment (e.g., reduced empathy and guilt) and behavioral disturbance (e.g., criminal activity) (Hare, 1991). Of particular importance to the current study, psychopathy is considered to be one of the prototypical disorders associated with empathic dysfunction, an absence of the appropriate empathic response to the suffering of another (Aniskiewicz, 1979; Hare, 1991).

The psychopath’s lack of affective empathy plays an important role in moral reasoning. Many studies support a dual-process model of moral judgment (Greene et al., 2008), in which both automatic emotional processes and controlled cognitive processes drive moral judgment. According to this theory, some moral judgments are driven primarily by social-emotional responses, while other moral judgments are driven less by social-emotional responses and more by cognitive processes (Greene et al., 2004).

Automatic emotional processes normally dominate for deontological decisions, while controlled cognitive processes drive utilitarian decisions (Duke and Bègue, 2015). This distinction is evident in moral dilemmas; a prototypical utilitarian favors performing actions in the name of the greater good, while a prototypical deontologist regards this actions as an unacceptable violation of rights and duties (Greene et al., 2008).

One such moral dilemma is the footbridge dilemma, in which a trolley threatens to kill five people, who can only be saved if you decide to push a stranger off the bridge, onto the tracks below. The stranger will die if you push him, but in the process, his body will prevent the trolley killing the five others (Thomson, 1985). Automatic emotional responses tend to drive people to disapprove of pushing the man off the footbridge, while controlled cognitive processes tend to drive people to approve of this action (Greene et al., 2008). Normally, in this particular dilemma, the automatic emotional response prevails; most people do not decide to push the man off the bridge (Greene et al., 2001). However, in the case of psychopathy, one would expect psychopaths to push the man given their lack of empathic concern. Studies indeed show that psychopathic personality characteristics, especially decreased levels of empathy, correlate with utilitarian choices (e.g., Bartels and Pizarro, 2011; Conway and Gawronski, 2013; Gleichgerrcht and Young, 2013).

Although it’s relatively clear that there’s a strong relationship between empathic concern and utilitarian reasoning, studies that actually measure the utilitarian reasoning of psychopaths are very scarce. In a recent study by Koenigs et al. (2012), psychopathic and non-psychopathic participants made judgments on 24 moral dilemmas. Results indicated that across all moral scenarios, psychopaths endorsed a significantly greater proportion of the proposed utilitarian actions than did the non-psychopaths. However, another recent study found no differences in utilitarian moral judgment between psychopaths and non-psychopaths (Cima et al., 2010). This lack of significant differences could be attributed to the smaller sample size and more lenient criteria for classifying participants as psychopaths (Koenigs et al., 2012).

These studies present participants with a variety of moral dilemmas, which can be distinguished by the extent to which the dilemma engages cognitive and affective processes respectively (Greene et al., 2001). The footbridge dilemma is considered a “personal dilemma”; it involves direct, intimate, physical contact (Greene et al., 2004). This type of dilemma engages emotional processing to a greater extent than other dilemmas (Greene et al., 2001). Previous studies show that some personal dilemmas, such as the footbridge dilemma, can be considered relatively easy (Wiegmann et al., 2013), while others can be considered more difficult; the latter bring cognitive and emotional factors into a very balanced tension. An example of a difficult personal dilemma is the crying baby dilemma, in which participants must decide whether it is appropriate to smother a child in order to save oneself and other townspeople. In response to this dilemma, participants tend to answer more slowly and show less consensus.

In contrast to personal moral dilemmas, there are also impersonal dilemmas that involve more indirect, remote actions or rule violations (Greene et al., 2004) and engage emotional processing to a lesser extent (Greene et al., 2001). A classic example of an impersonal moral dilemma is the standard trolley dilemma (Foot, 1978), in which a runaway trolley is approaching five railway workmen and the only way to avoid their deaths is to hit a switch that will cause the trolley to change the path and kill one single workman instead.

Many previous studies have shown that *personal* moral dilemmas, like the footbridge dilemma, elicit increased activity in brain regions associated with emotion and social cognition (Greene et al., 2001). Mendez et al. (2005) found that patients with frontotemporal dementia, who are also known for their emotional blunting, were disproportionately likely to approve of the action in the footbridge dilemma. Koenigs et al. (2007) found similar results studying patients with emotional deficits due to ventromedial prefrontal lesions. A recent study by Koenigs et al. (2012) showed that only low-anxious psychopaths were significantly more likely to endorse personal harms in moral dilemmas. Compared to non-psychopaths, both types of psychopaths were significantly more likely to endorse the impersonal actions. The differences between low and high anxious psychopaths are less relevant to our study, but the findings of Koenigs et al. (2012) show that, in order to be thorough, this study should measure reasoning in both personal and impersonal dilemmas.

Hence, in our study, we will ask participants to complete three types of moral dilemmas: an easy personal (the footbridge dilemma), a difficult personal (the crying baby dilemma) and an impersonal (the standard trolley dilemma) dilemma; these tasks will form part of our dependent variables. At the start of the study, participants will be presented with one explanation regarding why psychopaths exhibit the low levels of empathy required to make utilitarian choices in these tasks. Importantly, only one of these explanations will refer to the neurobiological features of psychopathy in order that we can isolate the effect of making a biological attribution for the behavior. We will now consider, more broadly, the effect of describing mental conditions in biological terms.

### Biological Attributions

Belief in biological explanations of behavior affects the perception of people suffering from a number of psychiatric disorders (Hyman, 2007). The comprehension of biological explanations of mental illnesses depends on the lay solution to the dualistic mind-body problem (Kendler, 2005). This raises the question of how lay people might view the brain relative to the mind and how this could influence the inferences that are drawn from neuroscience.

The effects of biological attributions represent a double-edged sword (Aspinwall et al., 2012). On one hand, biological explanations can have positive effects on lay conceptions of mental disorder. If the disorder is deemed biological, people may view sufferers of the disorder as less responsible for having the disorder, thereby blaming and stigmatizing sufferers to a lesser extent (Corrigan and Watson, 2004). On the other hand, biological attributions may also have negative effects; a biological disorder may be viewed as less changeable, also as a result of the perception that biological causes are uncontrollable. Consequently, patients, their families and friends may be less likely to believe in the efficacy of treatment, thereby reducing any placebo effect of treatment (Angermeyer et al., 2011). Hence biological attributions represent a double-edged sword.

Lebowitz (2014) reviewed studies assessing the impact of biological explanations of mental illnesses. Observational studies indicate that individuals who ascribe their mental illness to biological causes are more pessimistic about the success of their treatment. Moreover, belief in biological explanations was often related to greater stigmatization, given the perception that biological disorders are unchangeable. In contrast, experimental studies suggest that pessimistic views about the success of treatment are reduced when people receive information about the changeability of biological components of illnesses. However, individuals who have a mental illness and believe in a biological explanation of that illness do not show reduced compliance with treatment programs (Lebowitz, 2014). Consequently, belief in biological explanation has an impact on how lay people perceive their own psychiatric disorders and on others, thus having an effect on perceived blame for the condition and thereby potentially influencing treatment success.

### Advances in Science Communication

Today neuroscience appears to be particularly popular in the public eye as a means of explaining behavior. Indeed, evidence suggests that people find explanations of behavior more persuasive if those explanations feature circular references to the brain (Weisberg et al., 2008; Fernandez-Duque et al., 2015). Given its capacity to explain multiple aspects of the mind in a seemingly objective way, people have increasingly sought neuroscientific explanations of complex behaviors (Satel and Lilienfeld, 2015). The term ‘neuromania’ describes the tendency of the public to place greater faith in psychological explanations that are supplemented with references to the brain (Legrenzi and UmiltaÌ, 2011). Given its power to draw attention to scientific explanations of behavior, neuroscience could indeed be presented in various professional settings, such as the criminal justice system.

In this regard, Greene and Cohen (2004) predict that neurobiological explanations of criminal behavior will, and should, change lay attributions of free will and moral responsibility to offenders by rendering the physical mechanisms of human behavior more visible. Indeed, our increasing knowledge of the behavioral consequences of deficits in brain regions implicated in decision-making, morality and empathy may 1 day be integrated into the criminal justice system (Umbach et al., 2015). In accordance with such reasoning, we believe that in the future, the criminal justice system will be informed by science that is far more advanced than currently exists. Specifically, we predict that one day offenders may receive direct personalized feedback regarding the presence or absence of cognitive, genetic and neurobiological predictors of different mental illnesses and criminal behaviors. This may be useful in multiple contexts, such as prior to receiving a sentence in court and upon entering and departing prison grounds. For instance, criminal psychopaths could be shown how certain parts of their brain, specifically the limbic structures, exhibit less affect-related activity (Kiehl et al., 2001). Such procedures would grant offenders an understanding of the otherwise hidden scientific reasons behind their criminal behavior.

Given the practical and ethical issues implicated in measuring the response of real offenders to personalized scientific feedback, in our study, we are interested in analyzing how lay people respond to such feedback. In the current age of technology, social media has generated major new opportunities to analyze behavior online; in particular, by capturing the so-called ‘digital footprints’ left by millions of people on social networks. Using these sources of big data, researchers are generating opportunities for people to receive personalized data-driven feedback about their psychological and physical health. For example, Kosinski et al. (2015) analyzed the data of millions of Facebook users to create an algorithm capable of predicting users’ gender, sexuality, age, personal interests and political views, only based on their Facebook profiles (including statuses, likes, etc). Such algorithms have also been used to identify the possible psychopathic traits of ordinary people (Garcia and Sikström, 2014).

The method of the current study is based on this idea that trait information can be inferred from an individual’s Facebook profile. Specifically, participants will be given false feedback about having high or low psychopathic traits after entering their Facebook login details; the effect of providing such feedback on their moral behavior will then be measured. If individual scientific feedback is capable of changing the moral behavior of lay people, one might also expect this feedback to influence the moral behavior of offenders who receive such feedback in the future. Hence the findings of our study will pose implications for the real world, in which personalized neuroscience might one day influence how offenders are treated after trial, how offenders explain their own criminal behavior and therefore their own likelihood of reoffending (Maruna and Copes, 2005).

### The Impact of Belief in Free Will on Behavior

Previous research has shown that attributions of free will can influence behavior on many different levels: studies have documented effects of belief in free will versus disbelief in free will on well-being (Crescioni et al., 2015), self-control (Rigoni et al., 2012), cheating (Vohs and Schooler, 2008), aggression (Baumeister et al., 2009) or conformity (Alquist et al., 2013). Therefore, belief in free will poses important implications for how people behave. Hence, we will begin this section by considering the behavioral consequences of adopting different perspectives on the causes of behavior, where neuroscience could induce a change in such perspectives.

### Mindsets

In order to contextualize the hypothesized effects of attributions for psychopathy, we draw upon the analogy of attributions for intelligence, which have received far more empirical attention. In the study of intelligence, two different views about the nature of intelligence have emerged: the view of intelligence as a fixed part of a person’s personality that cannot be changed, and the view of intelligence as incremental (i.e., as always having the potential to be improved through exercise and effort). Dweck (1999) labeled these implicit theories as ‘growth’ and ‘fixed’ mindsets (or incremental theory and entity theory), and applied these theories to her research in self-theories, motivation, and personality.

A growth mindset refers to the belief that a person’s abilities are not predetermined, but can develop, improve and change over time through practice. In contrast, the ‘fixed mindset’ implies that a person’s abilities are static and cannot be changed as they are predetermined. These terms can be linked to the concepts of determinism and free will: a growth mindset implies the potential to change through the exercise of free will or a change in environments, whereas a fixed mindset implies belief in genetic and fatalistic determinism, such that any conscious motivation to change is futile.

Whether people believe in growth or fixed mindsets poses important implications for their behavior: inducing a growth mindset as compared to a fixed mindset greatly influences people’s levels of intrinsic motivation (Dweck, 1999, 2006). Dweck’s studies indicated, for example, that people who learnt about growth mindsets reacted in a far more positive way to failures than people who were taught about fixed mindsets. While those with a belief in the growth mindset used their failure as a reason and motivation to improve in the future, those with a belief in a fixed mindset reacted in a much more negative way. Specifically, those with the fixed mindset belief blamed others for their failure, made excuses or even became depressed; as they believed that their abilities were predetermined and could not be changed over time. Hence, it appears that the way in which people respond to feedback about their learning depends on the extent to which they perceive intelligence to be controllable.

Similarly, we hypothesize that the way in which people respond to feedback about their psychopathic traits depends on the extent to which they perceive psychopathic traits to be controllable. Participants will read either a neurobiological or a cognitive description of their psychopathic traits. We hypothesize that the neurobiological explanation of psychopathy will undermine the perception that psychopathic traits are controllable and therefore undermine the perceived moral responsibility of the participant. In the terms of Dweck (1999), we expect the neurobiological and cognitive attributions respectively to promote a fixed (uncontrollable) and growth (controllable) mindset toward psychopathic traits.

The effect of neuroscience communication on attributions of control and moral responsibility to the self has yet to be tested. Hence our predictions are based on the emerging body of research that considers the impact of neuroscience communication on attributions of moral responsibility to other people. Specifically, researchers have tested the effect of describing mental illnesses in neurobiological terms on the attributions of moral responsibility to criminal behaviors that are related to those illnesses. In mock court scenarios, people attribute less moral responsibility to an offender whose mental illness is described in neurobiological, rather than solely cognitive, terms (Gurley and Marcus, 2008; Greene and Cahill, 2011; Schweitzer et al., 2011; Schweitzer and Saks, 2011; Aspinwall et al., 2012).

The net mitigating effect of neuroscience has also been found with real judges engaged in mock sentencing (Aspinwall et al., 2012) and real sentencing (Denno, 2015). Similarly, students recommend shorter prison sentences for a mock offender after taking a cognitive neuroscience module and after reading an article about brain stimulation or the neuroscientific predictors of conscious intent (Shariff et al., 2014). Collectively this research lends support to Greene and Cohen’s (2004) prediction: people may recognize neurobiological dispositions to offend as undermining the culpability of offenders and their deservingness of punishment, unlike social dispositions to offend (Dar-Nimrod and Heine, 2011).

Researchers have considered the effects of presenting neuroscience not only as an explanation for the mental illness inflicting a particular defendant but also as a complete explanation of all behaviors in general (Nahmias et al., 2007). In this context, far fewer participants believed that people had free will (and could be held responsible) in the neurobiologically (relative to cognitively) determined world (38% vs. 85%, excluding responses of ‘I don’t know’).

As replicated by Nahmias et al. (2005), the vast majority of participants continued to attribute responsibility to the cognitively determined actor, thereby demonstrating a ‘compatibilist’ perspective on free will: the philosophical position that people are morally responsible for their actions even if those actions are the inevitable outcome of a chain of preceding events (Kane, 1999). Hence, neuroscience may challenge belief in free will not by highlighting the chain of preceding causal events but by suggesting that, as a neurobiological phenomenon, the cause of behavior must be somewhat unconscious; somewhat beyond the control of conscious thought. This dualist perception of neurobiological phenomena as unconscious might grant neurobiological determinism greater opportunity to challenge belief in free will than cognitive determinism. In other words, neuroscience might challenge attributions of responsibility by reducing the perceived availability or causality of conscious cognition rather than by promoting belief in determinism. In respect to our study, therefore, we expect the neurobiological explanation of psychopathy to reduce belief in free will to a greater extent than can be explained by any corresponding increase in the acceptance of determinism.

Regardless of the mechanism, the findings of Nahmias et al. (2007) suggest that, for judgments of people in general, neurobiological causation is granted more exculpatory power than conscious causation. Our study seeks to extend this finding to perceptions of the self in particular, rather than people in general, by applying the theory of fixed and growth mindsets beyond attributions for intelligence to attributions for psychopathic traits. First, we expect a neurobiological explanation of psychopathy to promote a fixed mindset toward psychopathic traits; a perception of psychopathic traits as uncontrollable, unchangeable and therefore beyond the moral responsibility of the individual. Second and in contrast, we expect a cognitive explanation of psychopathy to promote a growth mindset toward psychopathic traits; a perception of psychopathic traits as controllable, changeable and therefore within the moral responsibility of the individual. In order to support the hypothesis that neuroscience will reduce attributions of moral responsibility, we will now consider a proposed mediator of this relationship; that is the effect of neuroscience communication on how the mind and brain are perceived to relate to each other.

### Dualism

Dualism and physicalism are the two opposing philosophical solutions to the problem of how the mind and the brain are connected. Dualism corresponds to the belief that mind and brain are separate, whereas physicalism assumes that the subjective experience of humans is a function of brain activity. Forstmann and Burgmer (2015) found that adults intuitively believe in mind-body dualism and that dualism is the default mindset of lay people. In the current study, we are interested in how communicating neuroscience might influence this default mindset and behaviors that are affected by dualist intuitions. Since neuroscientific explanations of human behavior assume that our thinking and thus the mind are represented in the brain, we predict that neuroscience communication could challenge intuitive lay belief in dualism.

There is some evidence that whether people believe in physicalism or dualism poses implications for their choices in real-life. Specifically, Forstmann et al. (2012) considered the impact of dualist beliefs on health behaviors: participants who were primed with dualistic beliefs reported less commitment to healthy behaviors and made less healthy real-life decisions compared to participants primed with physicalism. Although Forstmann et al. (2012) observed that priming physicalist beliefs promoted healthy behaviors, we predict that physicalism would actually promote immoral behavior. Their study only documents the effect of dualism on health behaviors rather than moral behaviors: eating unhealthy food does not represent an act of aggression toward oneself and choosing a healthy lunch is not a moral behavior even though it has implications for one’s well-being. Forstmann et al. (2012) reason that physicalist beliefs promote health behaviors through their implication that the state of the body influences the state of the mind. We do not expect physicalist beliefs to promote moral behaviors in this way, since the behaviors measured in our study – cheating and utilitarian reasoning – bear no implications for bodily health.

Nevertheless, there is another mechanism by which physicalist beliefs might influence moral behavior. This mechanism concerns the potential relationship between dualistic beliefs and belief in free will, where the latter has been found to influence various forms of behavior linked to morality; those are self-control (e.g., Rigoni et al., 2012), cheating (e.g., Vohs and Schooler, 2008), aggression (e.g., Baumeister et al., 2009) and conformity (Alquist et al., 2013).

There are two mechanisms by which physicalist beliefs could challenge belief in free will. First, the perception of the mind as brain activity might highlight the causal chain of events that generates any behavior: people may more readily represent brain activity as a closed loop, in which present brain activity is the necessary and sufficient result of preceding brain activity in an unbreakable and inevitable chain of events. In contrast, people may more readily represent mental activity, in which present thoughts are not the necessary and sufficient result of previous thoughts. In other words, physicalism, as promoted by neuroscience, may illustrate the philosophy of determinism more effectively than the perception of mental activity independent of brain activity. Second, the perception of the mind as brain activity might bolster the belief that the mind – or cognitive influences on behavior – are largely unconscious and therefore beyond the control of conscious thought. Given the perceived compatibility of cognitive, yet not neurobiological, determinism with free will (Nahmias et al., 2005), we predict the second mechanism to constitute the means by which dualistic beliefs are reduced in the current study.

In their study, Forstmann et al. (2012) report preliminary data indicating that measures of mind-body dualism, free will and determinism are largely uncorrelated. We find this result most surprising and in fact predict a positive relationship between beliefs in dualism and free will. If the brain is conceived to constitute the mind, causal influences may subsequently appear to exert their effects beneath the scrutiny of conscious awareness. Hence we expect belief in physicalism to undermine belief in free will. Likewise, ‘libertarian views about free will [, that is belief in an independent free will, are]…likely rooted in some kind of dualism about mind (or soul) and brain’ (Kolber, 2016, p. 8). Therefore, we conclude that neuroscience could promote immoral behavior by undermining lay belief in dualism, the causal contribution of conscious thought and consequently free will; hence, we now consider the effects of belief in free will on immoral behavior.

### Cheating

In initiating this line of research, Vohs and Schooler (2008) investigated the relationship between belief in determinism and cheating behavior. As hypothesized, reading a passage on neurobiological determinism and the non-existence of free will by Crick (1994) led to a significant increase in cheating as compared to the control group. The findings were replicated in a second study that measured a more proactive form of cheating. However, the results failed to replicate in a third study that was part of the collaborative ‘Estimating the Reproducibility of Psychological Science’ project (Open Science Collaboration, 2015).

While cheating will also be measured in our study, we intend to use a far less explicit means of manipulating belief in free will than previous research. Specifically, we intend to manipulate belief in free will by giving participants either a neurobiological or a cognitive explanation of psychopathic traits. This approach extends beyond previous research by separating the two phenomena of determinism and free will rather than conflating them, as was common in previous manipulations (e.g., Crick, 1994). The manipulation in our study is also more representative of the means by which lay belief in free will could be challenged in the future. People will arguably be informed increasingly about neuroscience not only in the media but also in the use of neuroimaging. This could help to inform individuals about their neurobiological health and to modify brain states using neurofeedback and brain stimulation.

There is also reason to believe that people will be persuaded more by neuroscience than the personalized cognitive feedback that they receive from self-assessment questionnaires today and the philosophical arguments presented in previous research (Greene and Cohen, 2004).

In fact, studies have shown that psychological information appears to be more appealing and salient if accompanied by additional, and frequently superfluous or irrelevant, neuroscientific explanations (Weisberg et al., 2008). This neuroscientific bias is due to lay theories and reverence for the natural sciences that consequently are regarded more than social science explanations (Fernandez-Duque et al., 2015).

The current study measures cheating using the ‘die-under-cup’ task (taken from Shalvi et al., 2012), where people can reap benefits by misreporting the outcome of a die roll. Certain factors including number of times the die is rolled, the outcomes of other rolls, and time pressure, have been shown to increase dishonesty in this die-roll test (Shalvi et al., 2011, 2012; Gino and Ariely, 2012; Lewis et al., 2012). For our research, the die-under-cup paradigm is adapted to suit into an online questionnaire and to include conditions that increase likelihood of dishonesty.

### Aggression and Helpfulness

Baumeister et al. (2009) investigated Vohs and Schooler’s (2008) findings further by assessing the effects of belief/disbelief in free will on pro- and anti-social behavior in three experiments. In their research, disbelief in free will increased aggression and reduced helpfulness, while belief in free will resulted in more pro-social behavior such as the willingness to help. One might speculate therefore that promoting belief in free will generates a greater sense of personal responsibility and accountability for one’s actions, which arguably promotes socially desirable behavior. The finding that belief in free will motivates pro-social behavior is particularly relevant to our research, since we will test the effects of communicating neuroscientific explanations of psychopathy on the moral behavior of lay people.

### Self-control

Theoretically, telling a person that free will does not exist (directly or indirectly) could lead to that person being less willing or able to exercise self-control, which might actually explain the effects of disbelief in free will on cheating and aggression. If you believe that you can not control your life in any ultimate way, you may feel that there is no point in trying to control each of your actions, including impulses to act immorally. Several studies now confirm the idea that belief in free will is linked to self-control, both when operationalized at the levels of conscious perceptions and preconscious neural activity. In one study, weakening belief in free will reduced both perceived self-control and intentional inhibition (Rigoni et al., 2012). The authors interpreted these results as indicating that reduced self-control could be the mechanism by which disbelief in free will leads to antisocial tendencies.

The finding that disbelief in free will reduces self-control has also been documented at the level of basic neurocognitive processes. In one study, inducing disbelief in free will attenuated neural reactions to error, which are implicated in the very early phases of exerting self-control (Rigoni et al., 2015). Moreover, brain correlates of preconscious motor preparation were shown in the first study to be altered by inducing a belief in determinism, as compared to a belief in free will (Rigoni et al., 2012). In the context of the current study, self-control at the behavioral level will be included as a potential mechanism by which the manipulation influences moral reasoning and cheating.

### Conformity

While disbelief in free will may reduce self-control, it may increase social control; that is the influence that other people have on the behavior one exhibits. Indeed, research by Alquist et al. (2013) has shown that independently, less belief in free will and greater belief in determinism resulted in greater conformity to the judgments of other participants. It was suggested that a belief in free will contributes to more autonomous decisions and actions and therefore less conformity (to group norms).

This finding bears relevance to our online study, since participants will be provided with a supposedly scientific judgment about their degree of psychopathic traits. Different participants may conform to this judgment of themselves to differing extents; some participants may exhibit the psychopathic tendencies that they are described as having, while others may not. Given its expected effect on belief in free will, the neurobiological explanation of psychopathy might promote the conformity of participants to the psychopathy feedback. In contrast, since we do not expect the cognitive explanation of psychopathy to challenge belief in free will, participants who read this explanation may conform less to the feedback about their degree of psychopathy. Therefore, by reducing belief in free will, neuroscience may increase the receptivity of participants to external opinions, including the personalized science that we present. Hence, the persuasiveness of the opinion represents an additional factor that could explain the greater effect of neuroscience. We intend to capture and control for this effect by measuring the perceived believability of the presented explanations of psychopathy; the neurobiological explanation is hypothesized to be more believable.

### Summary and Hypotheses

Considering all of the above, there are three ways in which our study will add to the literature in this field. First, we will be testing the effects of specifically presenting neuroscience to lay people, rather than a generic passage about free will and determinism (e.g., Vohs and Schooler, 2008). Second, we will be looking at the effects of presenting neuroscience to explain a particular set of traits – psychopathic traits – among lay people rather than presenting explanations of a mental illness in a clinical population (see Lebowitz, 2014). Third, our study will examine the effects of providing personal feedback about psychopathic traits that was allegedly generated from a digital footprint (i.e., Facebook ‘likes’) rather than a survey measure of psychopathy.

The field study and the online study bear relations to each other, since our field study will test whether the public are sensitive to the hypotheses we propose for the online study. While the online study tests how the communication of the basis of psychopathy affects moral behavior, the field study is intended to capture the general public’s attitudes toward this communication. This will be done using the lost-letter technique, comparing return rates of postcards describing neurobiological or cognitive explanations of criminal behavior intended for prisoners or non-prisoners. We hypothesize that people will be sensitive to the potential negative behavioral consequences of communicating a neurobiological explanation of criminal behavior, as reflected by reduced return rates of the postcards. Specifically, we predict the return rate, indicating endorsement of the postcard’s content, to be higher for the cognitive (than neurobiological) explanation (Hypothesis 1) and higher in the non-prisoner (than the prisoner) condition (Hypothesis 2), and that these effects will interact (Hypothesis 3). Lay people may anticipate that neurobiological explanations of behavior undermine attributions of responsibility and hence seek to avoid the communication of neuroscience to offenders.

In comparison, the online study will measure whether this anticipation is justified; specifically, whether feedback about the neurobiological or cognitive psychopathic traits (specifically the strength of their moral alarm) of the participant influences utilitarian reasoning in moral dilemmas and dishonesty in a die-under-cup test, and whether this is mediated by self-control, and beliefs in dualism, free will and determinism. We hypothesize that participants who are led to believe they have a weak moral alarm (associated with higher levels of psychopathy) will act in ways consistent with psychopathic tendencies, i.e., use more utilitarian reasoning and cheat more (Hypothesis 4), especially after reading a neurobiological explanation of psychopathy (Hypothesis 5). Our final hypothesis (Hypothesis 6) is that our manipulation will influence self-control and belief in dualism, free will, and determinism, and that these will mediate the relationships outlined in Hypotheses 4 and 5.

## Study 1 – Field Study

### Materials and Equipments

#### The Lost Letter Technique

The lost letter technique (LLT) was first adopted by Merritt and Fowler (1948) as a means of assessing the public’s attitudinal approach to an undelivered letter (Stern and Faber, 1997). By distributing a large number of apparently lost letters referring to a particular topic, the return rate of the letters can be used to measure the public’s compliance with such issue (Milgram et al., 1965). This method has been deemed as valid and can be implemented conveniently: participants are unaware of their participation in this unique sociological survey, whereby natural behaviors are recorded, possibly reflecting concrete attitudes (Milgram et al., 1965; Cahill and Sherrets, 1979). This technique will be used to evaluate the public’s approval of disseminating the neuroscience of criminal behavior to both lay people and prisoners.

### Stepwise Procedures

#### Participants

Data will be collected from the responses given by a convenience sample of participants, whereby no recruitment or selection criteria is required. Therefore, age and gender and other individual factors cannot be selected. Those who decide to pick up a lost postcard and either mail, ignore or purposely destroy it will be considered participants (Milgram et al., 1965). As this field study is non-obtrusive, the number of participants taking part in the study cannot be determined. However, 832 postcards will be scattered around the city streets, thus authorizing approximately the same number of people to unconsciously take part in the study. This sample size is sufficiently large given the moderate response rates recorded by prior research; for example, from 37% in poorer neighborhoods to 87% in richer neighborhoods (Holland et al., 2012).

#### Ethics Statement

The study has been approved by the Ethics Committee of the University of Oxford, and is fully compliant with the Declaration of Helsinki.

#### Design and Procedure

A total of 832 printed, stamped and addressed postcards will be dropped throughout London. This large number of postcards will be dropped to increase the probability of gathering a large number of participants, thus increasing the sensitivity of the measure to the independent variable and the reliability of the obtained results (Milgram, 1969; Cherulnik, 1975). The postcards will be distributed face-up in proximity of parked cars, in shops and on pavements throughout a random selection of London boroughs.

#### Boroughs of London

The 832 postcards will be distributed in boroughs of London with different socio-economic status (SES) by four members of the research team. The SES of the borough will be calculated from the combined average degree of inequality, homelessness, housing quality, unemployment, income, benefits, and education (Trust for London and New Policy Institute, 2017). Within each of the four categories of SES, postcards will be distributed in the following boroughs:

Poorest: Barking and Dagenham, Newham, Brent, Ealing
Poor: Enfield, Haringey, Waltham Forest, Lewisham
Rich: Hackney, Southwark, Tower Hamlets, Croydon
Richest: Islington, Lambeth, Camden, Kensington and Chelsea, Merton

#### Distribution Process

Two hundred and eight postcards will be distributed in the poorest, 208 in poor, 208 in rich, and 208 in the richest boroughs. This decision has been made with the aim of reducing the probability of reaching a floor or ceiling effect of the manipulation: if the return rate is already high or low as a result of the borough SES (Holland et al., 2012; **Table 1B**; Kraus and Keltner, 2013), the scope for our manipulation to exert a supplementary effect may be limited. Therefore, we will distribute the postcards in boroughs of differing SES to ensure there remains this scope for the manipulation, while also increasing the generalisability of the findings from a more representative sample of boroughs.

The distribution will take place on 4 days (Monday, Tuesday, Thursday, and Friday) across four different time slots, such that each of the distributors will drop 208 postcards (52 per day of all four types of postcards). Each distributor will rotate through the four conditions, such that postcard type B is dropped after type A, C after B, D after C, A after D etc. On Monday the distribution will take place at 9–11 am, on Tuesday at 11 am-1 pm, on Thursday at 1–3 pm and on Friday 3–5 pm. A day of distribution will, however, be skipped until the following weekday if it is raining, since rainy weather could severely reduce the response rate. The distributors will drop the postcards on the same days and at the same times so that any effects of external factors between boroughs (e.g., the weather, time of day) are minimal. Thus, one person will distribute the postcards in one of the poorest boroughs, the second person in a poor borough, the third person in a rich borough, and the fourth person in one of the richest boroughs. On the second day the first person will distribute the postcards in a poor borough, the second person in a rich borough, the third person in one of the richest boroughs, and the fourth person in one of the poorest boroughs etc. Consequently, every distributor will drop postcards in every category of SES. No borough will be visited twice.

In the process of distribution the distributors will drop the postcards not too close to each other so that one person will not find two postcards. Furthermore, the distributors will drop the postcards in place that are visible and accessible to the general public so that the postcards can be found easily. In addition, the postcards will be dropped carefully and secretly.

#### Content

The postcards will be addressed to a PO BOX address (to avoid the use of a real traceable address), with the manipulation bolstered by supplementing the first line of this address with the ‘Organisation for Educating Prisoners/Students in Crime.’ All postcards will have the same front cover, as to avoid different images or colors biasing the participant’s subsequent response. An exception will be made for the front-cover wording, in so far as brain-based postcards will contain the emboldened word “brain,” and mind-based ones will contain the emboldened word “the person”. Thus far, research has documented only that adults do not perceive specific mental traits (e.g., memory) to be entirely physical (Forstmann and Burgmer, 2015). This suggests that people perceive ‘the person’ to consist of physical and non-physical causes of behavior. It remains possible, however, that the same people still equate the linguistic labels of ‘mind’ and ‘brain’. So people may be more dualistic in their implicit beliefs (when judging specific traits) than in their explicit beliefs (when judging ‘the mind’ as a concrete label). Since this study intended to test the effects of dualistic reasoning, the manipulation was designed to engage implicit beliefs about the person as a whole, rather than explicit beliefs about ‘the mind’. Hence the manipulation was oriented around ‘the person’. Therefore, this difference will guide subjects in understanding the explanations given in the postcard.

The main body of the postcard will comprise a brief description detailing the causes of criminal behavior, written by an imaginary person who has supposedly bought the postcard as part of a scientific campaign, the latter aiming to spread a particular message about the causes of criminal behavior. Half of the postcards will present a neurobiological (brain-based) explanation of criminal behavior (see Appendix), while the rest will present a cognitive (mind-based) explanation (see Appendix). Additionally, 50% of the postcards will be directed to prisoners (see Appendix), while the remaining half will be directed to non-prisoners (see Appendix). In both cases, the recipient will be an alleged friend of the writer. The writer will ask his friend to pass the postcard onto the ‘prisoners’ or (non-imprisoned) ‘students’ that he supposedly teaches. By comparing the response rates of all four conditions, one may infer different evaluations and conclusions about how neuroscientific and cognitive descriptions of criminal behavior influence the public’s decision to spread such information.

Consequently, participants will be randomly divided into four conditions, depending on the type of information and recipient reported on their postcard (**Table 1A**). As a result, a total of 208 postcards will be dispersed for each condition. This sample size was selected on the basis of power analysis assuming a normal distribution of the data (the power calculator we used can be found at https://www.stat.ubc.ca/~rollin/stats/ssize/n2.html). In this independent-samples analysis, we set the probability of Type I error to 5% and the probability of Type II error to 20%, and assumed that the effect size would be small (Cohen’s *d* = 0.2).

**Table 1A:** Conditions of the field study.

	Non-prisoner	Prisoner
Brain-based	208	208
Mind-based	208	208


**Table 1B:** Total number of dispersed postcards in London boroughs.

	Poor	Mildly poor	Mildly rich	Rich	Total
Brain-based to non-prisoner	52	52	52	52	208
Brain-based to prisoner	52	52	52	52	208
Mind-based to non-prisoner	52	52	52	52	208
Mind-based to prisoner	52	52	52	52	208
Total	208	208	208	208	832


#### Proposed Analysis and Anticipated Results

Statistical analysis will involve analyzing the response rates of all four conditions and so binary data (did not return the postcard = 0; returned the postcard = 1) will be collected. We expect to obtain an explanation effect supporting Hypothesis 1, by which a larger number of mind-based postcards than brain-based ones will be posted. A chi-square test of association will determine any significant differences between observed and expected response rates: χ^2^ (1, *N* = number of returned postcards) > 3.841, *p* < 0.05 (**Tables 2A**,**B**). Furthermore, an effect of the recipient is predicted, whereby we expect to receive a larger number of postcards addressed to non-prisoners than prisoner, supporting Hypothesis 2 (**Tables 3A**,**B**). A second chi-square test of association will be carried out to assess whether such difference in returned postcards exists: χ^2^ (1, *N* = number of returned postcards) > 3.841 *p* < 0.05. In particular, an interaction effect is expected, whereby we expect the effect of the recipient to be particularly strong for the brain-based postcards (Hypothesis 3). Therefore, we predict a larger difference between non-prisoners and prisoners in the brain-based condition than in the mind-based condition (**Tables 4A**,**B**). The software IBM SPSS Statistics Version 23.0 will be employed.

**Table 2A:** Expected frequencies of returned postcards due to given explanation.

Chi-square test of association: Expected frequencies (hypothetical *N* = 400) in returned mind-based and brain-based postcards			
	**Mind-based**	**Brain-based**	**Total**
Returned postcards	200	200	400


**Table 2B:** Observed frequencies of returned postcards.

Hypothetical Chi-square test of association: Observed frequencies (hypothetical *N* = 400) in returned mind-based and brain-based postcards			
	**Mind-based**	**Brain-based**	**Total**
Returned postcards	300	100	400


**Table 3A:** Expected frequencies of returned postcards due to receiver.

Hypothetical Chi-square test of association: Expected frequencies (hypothetical *N* = 400) in returned non-prisoner and prisoner addressed postcards			
	**Non-prisoner**	**Prisoner**	**Total**
Returned postcards	200	200	400


**Table 3B:** Observed frequencies of returned postcards due to receiver.

Hypothetical Chi-square test of association: Observed frequencies (hypothetical *N* = 400) in returned non-prisoner and prisoner addressed postcards			
	**Non-prisoner**	**Prisoner**	**Total**
Returned postcards	300	100	400


**Table 4A:** Interaction effect of expected frequencies of returned postcards.

Hypothetical Chi-square of contingency tables: Expected frequencies (hypothetical *N* = 400)			

	Non-prisoner	Prisoner	Total
Mind-based	100	100	200
Brain-based	100	100	200
Total	200	200	400


**Table 4B:** Interaction effect of observed frequencies of returned postcards.

Hypothetical Chi-square of contingency tables: Observed frequencies (hypothetical *N* = 400)			

	Non-prisoner	Prisoner	Total
Mind-based	215	85	300
Brain-based	85	15	100
Total	300	100	400


#### Limitations

The LLT has a number of limitations. First, one might question whether the technique is sufficiently sensitive to document subtle manipulations. For the manipulation to be successful, participants must attend to the address and text on the postcard and their decision to return the postcard (or not) must reflect their approval of this specific message. The participants, however, might not pay sufficient attention to the manipulation.

Second, even more so that the online study, the field study cannot document the mechanisms that mediate the decision to return the postcard. Whilst we interpret the return rate as indicating the degree of acceptance for the presented explanation of offending, the return rate will also be sensitive to unpredictable events (e.g., street cleaners who throw away different numbers of postcards of different conditions). We aim to overcome this problem by implementing a strict plan for dropping the postcards: each distributor will rotate between dropping cards from all four conditions in boroughs of every SES category at all dropping times, spread across the day (see distribution of postcards). Nevertheless, the LLT has been shown to be reliable (Milgram et al., 1965; Cherulnik, 1975). In addition, we have chosen to feature relatively extreme statements on the postcards in order to strengthen our manipulation. Thus this explicit manipulation may be strong, especially since participants will be unaware of their participation, thereby removing potentially overshadowing Hawthorne effects.

## Study 2 – Online Study

### Materials/Equipment

#### Neurobiological vs. Cognitive Manipulation

Our manipulation was adapted to focus on a neurobiological vs. cognitive understanding of psychopathy, based on a study by Aspinwall et al. (2012) where the explanation of psychopathy was drawn from James Blair’s neurocognitive model (Blair, 2006). We removed any direct references to genetics from the original stimuli to increase the scientific equivalence of the two explanations. The neurobiological details in the brain-based explanation were deliberately superfluous; in reality, these details contributed very little substance to the argument. This decision was based on findings that superfluous neuroscience increases the perceived credibility of psychological science, even when the neuroscience itself is a circular repetition of the psychological science (Weisberg et al., 2008). Here, we present the material for the two conditions in the same paragraphs, emphasizing the equivalence of the conditions independent of the manipulation:

##### The brain’s/mind’s moral alarm

Here, we present the material for the two conditions in the same paragraphs, emphasizing the equivalence of the conditions independent of the manipulation: Extensive research shows that human brains/minds have a moral alarm. The moral alarm is the physical/psychological system that produces feelings of anxiety when you behave badly. When humans behave badly, their brain/mind normally generates particular electrical signals and chemical reactions/thoughts and emotions that produce feelings of anxiety. The purpose of this anxiety is to physically/psychologically reduce your desire to behave badly.”

##### Your brain/mind

We would now like to tell you more about people like you, who have an 18-22% stronger/weaker moral alarm than the average person.

The moral alarm is the physical/psychological system in the brain/mind that produces feelings of anxiety when you behave badly. The purpose of this anxiety is to physically/psychologically reduce your desire to behave badly. Since your moral alarm is 18–22% stronger/weaker than the average moral alarm, you are 18-22% less/more likely to behave badly than the average person. This is true of anyone with an 18–22%stronger/weaker moral alarm.

People have moral alarms of different strengths because of physical/psychological differences in how their brains/minds work. When people with a brain/mind like yours behave badly, their brain/mind generates more/less of the electrical signals and chemical reactions/thoughts and emotions that produce feelings of anxiety.

Therefore, people with a brain/mind like yours feel 18–22% more/less anxious when they behave badly. Consequently, people with a brain/mind like yours are 18-22% less/more likely to behave badly.

#### The Short Dark Triad Scale

The Short Dark Triad scale (SD3; Jones and Paulhus, 2014) is a brief measure of three socially aversive traits – Machiavellianism, narcissism and psychopathy. The whole scale normally consists of 27 items, rated on a five-point scale from 1 (disagree strongly) to 5 (strongly agree). As we are only interested in one element of the dark triad constellation, psychopathy, we will only use the psychopathy subscale of this instrument. This subscale includes 9 items (e.g., “Payback needs to be quick and nasty”) and provides an efficient, valid and reliable way of measuring psychopathy, with Cronbach’s alpha ranging somewhere from 0.77 to 0.79 (Buckels et al., 2014; Jones and Paulhus, 2014). This scale will be used to assess the participants’ real psychopathic traits.

#### The Dualism Scale

We will measure dualistic beliefs with a modified version of the thought experiment used by Forstmann and Burgmer (2015). Participants are asked to imagine that scientists have developed a device capable of duplicating any person in a matter of seconds, using highly advanced technology. Participants are told that after placing a person into a chamber, a computer scans the entire person (i.e., the entire content of the chamber), its every molecule and atom, and stores the information digitally. The information is then used to recreate the scanned person from basic chemical elements in a second chamber, resulting in a 100% identical copy of the scanned object, with a 100% success rate. In contrast to the original task, our participants will be asked to imagine that they are placed in the first chamber and are duplicated. After the process is complete and a 100% perfect duplicate emerges, the participants will indicate on 7-point Likert-type scales ranging from ‘definitely no’ to ‘definitely yes’ the extent to which six properties of themselves also describe their duplicate. Three of the properties will be mental and relate to the manipulation text, e.g., “Is the moral alarm in your duplicate the same strength as the moral alarm in you?”. The remaining three items will be physical, e.g., “Does your duplicate have the same eye color as you?”. If people do separate minds from bodies, there will be a difference in the mental and physical properties ascribed to the duplicate.

#### The Determinism Subscale

The Determinism subscale of the Free Will Inventory (Nadelhoffer et al., 2014) consists of five items that make different deterministic statements. For example, “Every event that has ever occurred, including human decisions and actions, was completely determined by prior events.” Participants are asked to rate their agreement on a seven-point Likert rating scale with anchors ranging from 1 (strongly disagree) to 7 (strongly agree). The Determinism subscale has an acceptable to good internal consistency, with Cronbach’s α = 0.772 (Nadelhoffer et al., 2014).

#### The Free Will Subscale

The Free Will subscale of the Free Will Inventory (Nadelhoffer et al., 2014) consists of five items stating in different ways that free will exists. For example, one of the items states that “People ultimately have complete control over their decisions and their actions.” The scale was chosen over the FAD+ scale (Paulhus and Carey, 2011) because it avoids religious terms. Participants are asked to score each item on a scale from 1 (strongly disagree) to 7 (strongly agree). This subscale has a good internal consistency, with Cronbach’s α = 0.803 (Nadelhoffer et al., 2014).

#### Die-under-Cup Measure of Dishonesty

Dishonesty/cheating will be measured using an online version of the die-under-cup test (Shalvi et al., 2011). Participants will be asked to press the ‘next page’ button to roll a virtual die within the online questionnaire in place of the physical die and cup. The ‘die’ will be rolled three times, the results of which will be fixed to show a two, a six, and a three respectively. Participants will report the first outcome by typing the number into a box and this response must be made within a 30 s window. The die-under-the-cup appears to be a valid measure of dishonesty, as found in a study conducted by Halevy et al. (2013), whereby high scores on this task are caused by the participant cheating rather than luck.

#### Crying Baby Dilemma

The crying baby dilemma (Greene et al., 2001) involves participants deciding how to behave with their child when enemy soldiers have taken over their village. In order to save their own lives and every village townspeople’s lives, they must smother their crying baby to death, in order to avoid the attention of the enemy soldiers. Alternatively, saving the child would mean putting the whole village at risk and letting all townspeople face death.

#### Standard Trolley Dilemma

The standard trolley dilemma (Foot, 1978) involves the participant being at the wheel of a runaway trolley. The latter is approaching a track, at the end of which five railway workmen are standing. Participants are given the option to switch a lever on the dashboard so that the trolley proceeds off toward a right-hand track, where only one workman is standing. The participant is left to decide whether to take no responsibility for the situation and let the trolley proceed straight toward the five men, or change the trolley’s direction in order to save as many lives as possible.

#### Footbridge Dilemma

In the Footbridge dilemma (Thomson, 1985) a personal moral violation can be authorized in order to justify a precise utilitarian reasoning (Valdesolo and DeSteno, 2006). Individuals are presented with a scenario in which a trolley is moving toward five workmen who have no way to escape. The participants are asked to imagine that they are on a footbridge next to a large stranger, whom they may push off the bridge in order to stop the trolley from hitting the five workmen. By doing so, only one person would be actively killed and five people would be saved. Through a replication of the study by Greene et al. (2001), this task has been demonstrated to measure a different construct to personal moral dilemmas (Nakamura, 2013).

Utilitarian reasoning will be assessed by administering participants all three moral dilemmas.

### Stepwise Procedures

#### Participants

Participants will be recruited through adverts for the study posted on social media sites, such as Facebook. We will also contact universities in Austria, Germany, Italy, Slovenia, Finland, Norway, and the UK to promote the online study to their English-speaking students. Therefore, both students and members of the public aged 18 years of age or older will be able to take part in our study. The participants must be able to speak and comprehend English in order to fully understand all the information presented to them. Hence, we will ask participants to rate their English competence before completing the study. As we expect a small effect size, we aim to recruit at least 800 participants. This sample size was selected on the basis of power analysis. In this analysis, we set the probability of Type I error to 5% and the probability of Type II error to 20%, and assumed that the effect size would be small (Cohen’s *d* = 0.2). Upon completing the study, participants will be entered into a lottery, giving them the chance to win a sum of money ranging from €75 – €200.

#### Ethics Statement

The study has been submitted to the Ethics Committee of the University of Oxford, and is fully compliant with the Declaration of Helsinki.

#### Design and Procedure

**FIGURE 1:**
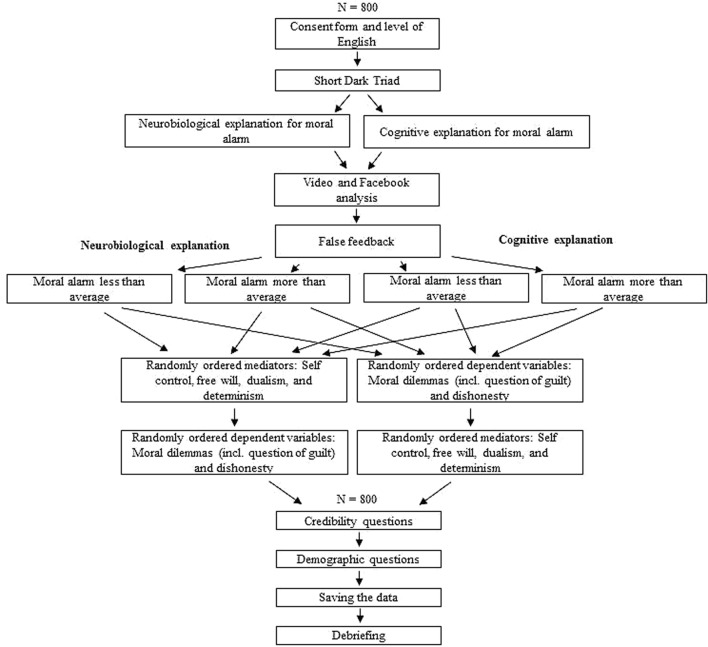
**Stepwise procedure of the online study**.

Participants will be asked to complete a series of online tasks in a single session (see **Figure 1**), administered via the Qualtrics platform. The online study will be divided into three sections, in order to facilitate the completion and understanding of the study. They will firstly be instructed that the purpose of this study is to investigate different ways of measuring personality traits, thus comparing traditional and newly developed means of measuring a certain psychopathic trait, specifically normal variation in the anxiety people feel when committing an immoral act. In addition, an abbreviated online version of the Short Dark Triad (SD3) will be administered to determine participants’ psychopathic traits. We will therefore be able to compare the influence of actual psychopathy with the influence of the false feedback about psychopathy presented to each participant.

In the second part of the online study, participants will be given either a cognitive or a neurobiological explanation of moral alarm; that is the anxiety produced during immoral behavior. This description will explain the function of the moral alarm in producing feelings of anxiety when people behave badly. A single multiple choice question will be included at the end of the description, in order to make sure that participants are reading thoroughly the online questionnaire and that our manipulations are effective. Participants will also be informed of why it is difficult to assess the moral alarm through a self-report questionnaire, thus justifying the purpose of the Facebook analysis. Again, a single multiple choice question will assess their understanding of the information given.

As a way of persuading participants that assessing moral alarm through online personal data is valid and reliable, they will all be asked to watch an online video reviewing Kosinski’s research (Kosinski et al., 2013) into the prediction of personality traits from digital footprints. The video will consider how researchers can predict personality traits, intelligence, ethnicity, political views and in particular psychopathic traits, by simply taking into account Facebook likes. A question measuring their accuracy in comprehending the text given to them will also be included. Accordingly, participants will be asked to provide a shortened link (through URL shortener) of their Facebook account for the purpose of analyzing their Facebook likes. None of the entered login details will be saved.

After entering their shortened URL to their Facebook page, participants will receive false feedback about their psychopathic traits. Note these traits will be described without actually referring to psychopathy in order to avoid triggering the popular negative perception of psychopathy. Specifically, participants will be randomly allocated to read one of four types of feedback: half of the participants will read that they have a 18–22% weaker-than-average moral alarm, while the other half will read that they have a 18–22% stronger-than-average moral alarm. Additionally, within each of these groups, half of the feedback messages will refer to a neurobiological (brain-based) explanation of moral alarm, while the remaining half will refer to a cognitive (mind-based) explanation of moral alarm. Both types of explanations were adapted from a subsection of the explanations presented by Aspinwall et al. (2012), who illustrated the power of biological explanations of psychopathic behavior, including moral alarm, to shape the sentencing decisions of judges. In sum, participants will read one of four different types of feedback that differ along two dimensions: the degree of personal moral alarm and the neurobiological or cognitive nature of this trait.

The third section of the study will require participants to complete a series of brief tasks. The measurements for the mediators and dependent variables will be counterbalanced. Therefore, half of the participants will complete the (randomly ordered) mediators, followed by the (randomly ordered) dependent variables. Additionally half of the participants will complete the (randomly ordered) dependent variables followed by the (randomly ordered) mediators. Consequently, the participants will complete scales intended to measure the proposed mediators, that is the Determinism Subscale, the Free Will Subscale of the Free Will Inventory, and a measure of dualistic beliefs.

Subsequently, self-control will be measured through a modified online version of the famous marshmallow test (Mischel et al., 1972). As all participants will be entered into a final lottery, they will be asked when they would prefer to discover the outcome of the draw. They will have the choice to either find out immediately after the completion of the study if they have won their specific amount of money, or whether they would prefer to receive an increment of €100 but wait 3 months to find out the lottery’s outcomes. Participants will be given the measure of belief in dualism. This measure concerns a futuristic device that enables scientists to precisely duplicate any person; participants are asked to answer questions about their hypothetical duplicate.

Participants will also be required to respond to three different moral dilemmas designed to measure utilitarian reasoning: the difficult personal dilemma, the easy personal dilemma and the impersonal dilemma. For the first type of dilemma, we will use the crying baby dilemma. The footbridge dilemma will be used to test the easy moral dilemma, while the standard trolley dilemma will be administered in order to test the impersonal dilemma. All three dilemmas will be counterbalanced, in order to avoid any first response interfering or influencing the remaining responses. At the end of each response, the participants will be asked whether they felt guilty about their virtual actions, through a 6-point Likert scale.

The die-under-the-cup test will also be administered to participants to measure their willingness to lie. They will be asked to press a button on the screen to roll a die three times to decide the amount of money they could potentially win. Finally, they will be asked to select the outcome of their first roll (from 1 to 6) on-screen; they will be given 30 s to enter the outcome before the page progresses. Participants will be warned that if they fail to type the outcome down within 30 s, they will only be awarded the minimum amount of money. The time limit will be visible from a ticking counter.

The roll outcome that participants report will determine the value of the lottery prize: the higher the outcome, the greater the value of the prize. Hence participants may misreport the outcome of their first roll in order to increase the value of their potential prize. In reality, the prize will be fixed at the maximum value. In order that participants can receive their potential prize for entry into the lottery, we will lastly ask for their email address. However, this email address will be stored separately to all other data to ensure their responses remain anonymous. Finally, participants will be debriefed about the false feedback.

It is important to note that a counterbalancing procedure will be included, whereby the tasks measuring self-control, dishonesty and utilitarian reasoning will be presented in a randomly generated order. This is important because we hypothesize that certain conditions, such as the stronger-than-average moral alarm condition, will promote more inhibitory, honest and empathic responses on the first measure of any psychopathic behavior. Consequently, this could reduce the *willingness* of participants to exhibit inhibitory, honest and empathic responses on subsequent measures of these behaviors.

According to moral licensing theory (Merritt et al., 2010), individuals who show moral behaviors initially, tend to display immoral, unethical or problematic behaviors later (Blanken et al., 2015). This may be attributed to the fact that such individuals feel authorized to award themselves moral credits, believe that all temptations wear down their self-control, or simply become desensitized to the thought of cheating. Participants in our stronger-than-average moral alarm condition may be more likely to cheat in later tasks than earlier task due to this confounding effect of moral licensing.

In contrast, we hypothesize that participants who read about their neurobiologically weak moral alarm may exhibit less inhibitory, less honest and less empathic responses during initial tasks, given the perception that their psychopathic traits are independent of their free will and are ultimately due to their brain. Consequently, the very act of behaving immorally may induce subsequent guilt and remorse, thereby reducing the perceived appropriateness of continuing to respond immorally. Therefore, participants may exhibit more moral behaviors in the later tasks.

For example, participants in the neurobiologically weak moral alarm condition who first receive the dishonesty task may feel that cheating is acceptable, given a reduced attribution of their actions to free will. However, participants may then believe that enough cheating has been done and therefore respond more morally in the subsequent tasks, such as the self-control one. In order to control all these possible outcomes, randomly changing the order of presentation of these tasks could minimize any possible confounding effects of completing each task on responses to subsequent tasks.

Before concluding the study, participants will be asked to what extent they thought the feedback they had received (i.e., the false feedback) was true about themselves. Furthermore, the participants will be asked to rate the degree to which they believed the presented explanation of psychopathy was true. Furthermore, participants will be asked to provide personal demographic information, including their age, gender, nationality and field of studies/work. At the end of the study, the participants will be comprehensively debriefed.

#### Proposed Analysis and Anticipated Results

We will test our hypotheses using hierarchical multiple linear regression, according to the recommendations of Hayes (2013). Hierarchical multiple linear regression is an appropriate procedure because we want to see how the average values of the dependent variables change as the independent variable is varied through our manipulation, while at the same time several demographic variables are held fixed. Hierarchical regression was selected instead of multivariate analysis of variance because we want to test hypotheses of mediation. Hayes’ procedure for mediation analyses involves bootstrapping confidence intervals of the indirect effects; this procedure was considered preferable over the “causal steps” model of Baron and Kenny (1986), due to several shortcomings of this model (for detailed coverage, see Hayes, 2013). The software IBM SPSS Statistics Version 23.0 will be employed. For the mediation analyses, the PROCESS macro for SPSS will be used^1^.

Before testing the model, we will check the assumptions of linear regression. If the observations are normally distributed, then parametric regression is appropriate. Outliers will be removed systematically. There will be no missing data points, as the form does not allow continuing without selecting an option. Nevertheless, participants may terminate their participation early: where participants discontinue their participation after at least one dependent variable has been measured and have not withdrawn their consent, the data will be used in the analysis of that particular variable. Categorical dependent variables will be dummy coded as whole numbers.

In the first step of the analysis, the dependent variables Dishonesty and Utilitarian reasoning 1 (Crying Baby), 2 (Footbridge) and 3 (Trolley) will be entered into the model. Then the independent variables will be entered in a fixed order of steps or blocks. In the first block of the hierarchical regression model, the demographic variables Age, Gender, Nationality and Education level will be included. This means that these variables are held constant in the further analyses. In the second block, the independent variables of Psychopathy and Neuroscience will be added, first separately and then together; the direct and interactive effects can be estimated in this manner. Next the mediation analysis of indirect effects through Free will, Determinism, Dualism, Guilt and Self-control will be carried out using the PROCESS macro.

We hypothesize that the dependent variables will be significantly predicted by the independent variables but also that the mediation analyses will show significant indirect effects. Specifically, in accordance with Hypothesis 4, we expect participants in the weak moral alarm condition to show more dishonesty and utilitarian reasoning compared to those in the strong moral alarm condition. We also expect to see a stronger demonstration of this in the neurobiological explanation condition (Hypothesis 5). Finally, we expect the indirect effects observed to support Hypothesis 6, and show that the measures of free will, dualism, guilt, and self-control mediate the relationships between the independent and dependent variables.

#### Limitations

The primary limitation of the online study will be our inability to identify the precise mechanism of the effects, e.g., which type of belief in free will has been challenged by the manipulation: a compatibilist or incompatibilist notion of choice? It is impossible to control for all the differences between the neurobiological and cognitive conditions. Specifically, the neurobiological and cognitive conditions might induce differences in lay perceptions of the availability and causal efficacy of the conscious mind over our feelings of moral alarm (compatibilist choice), or the scope for free will to exist before the brain/mind and therefore the scope to attribute ultimate control to our actions (incompatibilist choice). Given a more nuanced understanding of compatibilist choice, the neurobiological and cognitive conditions could also induce differences in the lay perception that the degree of moral alarm experienced is a feature of our Deep Self – our stable self – or merely our Acting Self – our temporary self in a particular situation (Sripada, 2009).

This limitation in our ability to specify the mechanism could only be overcome by measuring more mediators and including more control conditions, which would be impractical due to the number of participants and length of survey then required. Despite having no control condition in which participants perform all the tasks without reading about their own moral alarm, we can still establish effects of describing neurobiology relative to describing cognition – the purpose of our study. Our goal is to document effects of giving people personal feedback in neurobiological terms, not to document effects of giving people personal feedback relative to no feedback. Note, with this design, we can still document effects of giving above-average feedback relative to below-average feedback.

Given the nature of the manipulation, we can only recruit Facebook users for the online study. Although Facebook is very widely used, it is more popular among younger (and other types of) people. The cross-cultural design of our study, however, promotes the generalisability of our findings in a different direction: across the countries. In order to assess the generalisability of our sample, we are of course collecting demographic information in order to know if and how our sample could be biased.

One might also contest whether our findings can be generalized to real life examples of immoral behavior, since, for example, cheating was only measured online and people are more likely to lie online (Naquin et al., 2010). On the other hand, the potential for the researcher to record cheating is clearer online – this potential might therefore discourage cheating. Consequently, there is also reason to suggest the cheating observed online may not be any more frequent than the cheating observed face-to-face. One might also argue that measures of online cheating are gaining ecological validity with the increasing tendency for people to spend their time online.

The basis of our manipulation in a false analysis of Facebook Likes creates a potential pitfall for the credibility of the manipulation. Participants might not believe that we have analyzed their Likes and that their Likes reveal they have below/above-average levels of moral alarm. Also, the participants might suspect whether the cheating task is a genuine means of determining the amount of money available in the prize draw rather than a means of determining cheating. These potential artifacts will be monitored by asking participants about any suspicions and the believability of the manipulation at the end.

## Author Contributions

All authors listed have actively contributed to this work, and given approval for its publication.

## Conflict of Interest Statement

The authors declare that the research was conducted in the absence of any commercial or financial relationships that could be construed as a potential conflict of interest.

## Footnotes

1 processmacro.org
