# Supplementary material for: Communicating the Neuroscience of Psychopathy and Its Influence on Moral Behavior: Protocol of Two Experimental Studies
Source: Front Psychol. 2017 Mar 14;8:294. doi: 10.3389/fpsyg.2017.00294 (PMC5348490; doi:10.3389/fpsyg.2017.00294)
Supplement: Supplementary file 1 [file Data_Sheet_1.docx]

**APPENDIX**

**Postcard manipulation text**

**Neurobiological/Cognitive postcards (front)**

[Typed] Most criminal behaviour is completely caused by the failure of the person to resist impulses.

[Typed] Most criminal behaviour is completely caused by the failure of the brain to resist impulses.

**The neurobiological explanation for the prisoner (back)**

[Hand-written] Hi Alex,

I saw this and thought the prisoners you teach might be interested.

[Typed] Extensive research has discovered that most criminal behaviour is entirely the result of the failure of the person’s brain to resist impulses. Please take one of these postcards to help us spread the message to people who have committed crime.

[Hand-written] Sam

[Typed in small print] This postcard is part of a scientific campaign.

**The neurobiological explanation for the non-prisoner (back)**

[Hand-written] Hi Alex,

I saw this and thought the students you teach might be interested.

[Typed] Extensive research has discovered that most criminal behaviour is entirely the result of the failure of the person’s brain to resist impulses. Please take one of these postcards to help us spread the message to other people.

[Hand-written] Sam

[Typed in small print] This postcard is part of a scientific campaign.

**The cognitive explanation for the prisoner (back)**

[Hand-written] Hi Alex,

I saw this and thought the prisoners you teach might be interested.

[Typed] Extensive research has discovered that most criminal behaviour is entirely the result of the failure of the person to resist impulses. Please take one of these postcards to help us spread the message to people who have committed crime.

[Hand-written] Sam

[Typed in small print] This postcard is part of a scientific campaign.

**The cognitive explanation for the non-prisoner (back)**

[Hand-written] Hi Alex,

I saw this and thought the students you teach might be interested.

[Typed] Extensive research has discovered that most criminal behaviour is entirely the result of the failure of the person to resist impulses. Please take one of these postcards to help us spread the message to other people.

[Hand-written] Sam

[Typed in small print] This postcard is part of a scientific campaign.
